# Eine Frage der Erwartungen? Zur Qualitätsbestimmung von Wissenschaftskommunikation aus Publikumsperspektive

**DOI:** 10.1007/s11616-021-00701-z

**Published:** 2021-12-17

**Authors:** Nina Wicke

**Affiliations:** Institut für Kommunikationswissenschaft, TU Braunschweig, Bienroder Weg 97, 38106 Braunschweig, Deutschland

**Keywords:** Wissenschaftskommunikation, Qualität, Publikums- und Kommunikator*innenperspektive, Expert*innendebatte, Mixed Methods, Science communication, Quality, Audience and science communicators perspective, Expert debate, Mixed methods

## Abstract

Wissenschaftskommunikation trägt dazu bei, wissenschaftliches Wissen für die breite Öffentlichkeit zugänglich zu machen. Welche Ansprüche das Publikum hierbei an die Vermittlung richtet und inwiefern dies den Vorstellungen der Kommunikator*innen entspricht, wird bislang in Forschung und Praxis wenig berücksichtigt. Eine Auseinandersetzung mit Qualitätsfragen befindet sich in der Wissenschaftskommunikation noch in ihren Anfängen. Vor diesem Hintergrund wird auf der Grundlage des wissenschaftsjournalistischen Qualitätsdiskurses ein Vorschlag für mögliche Qualitätsdimensionen von Wissenschaftskommunikation entwickelt und am Beispiel des etablierten Formats der wissenschaftlichen Expert*innendebatte operationalisiert. Im Rahmen einer Pre-Post-Befragung erhebt die vorliegende Studie, orientiert an den Annahmen der Theorie der subjektiven Qualitätsauswahl von Wolling, Qualitätserwartungen und -bewertungen aus Publikumssicht. Die Perspektive der Formatverantwortlichen und was ihrer Ansicht nach die Qualität von Wissenschaftskommunikation kennzeichnet, wird kontrastierend in leitfadengestützten Expert*inneninterviews thematisiert.

Das Publikum erwartet insbesondere Glaubwürdigkeit, Zugänglichkeit, Unabhängigkeit, Neutralität und Vielfalt von dem Format und den debattierenden Expert*innen. Diese Dimensionen stellen gewissermaßen Grundvoraussetzungen dar, damit Teilnehmende den vermittelten Inhalten vertrauen und sie gegebenenfalls in ihrem Verhalten berücksichtigen können. Obwohl sich das Format in seiner Konzeption am Public Engagement-Paradigma orientiert, ist ihnen eine Einbindung weniger wichtig. Es zeigen sich Inkongruenzen zu dem, worauf die Kommunikator*innen beim Debattenformat Wert legen. Sie erachten im Unterschied zum Publikum eine hohe Themenaktualität und gesellschaftliche Relevanz als zentrale Qualitätsmerkmale. Das Spannungsfeld zwischen einem normativen Anspruch an Wissenschaftskommunikation und dessen Umsetzung in der Praxis spiegelt sich in ihren Aussagen wider.

## Abstract (en)

Science communication is considered important for individuals and is of high societal relevance, as it fosters a greater public understanding of science and scientific methods. It informs citizens, empowers them to form opinions, and allows them to access different topics and knowledge. To effectively fulfill these functions, science communication must be of high quality. However, the development of quality standards of science communication has so far received little attention in research and practice. Studies of audience expectations for science communication are scarce. Findings from journalism research suggest that there is a relationship between the audience’s perceptions of “good journalism” and their media use. If audiences have the feeling that their needs are not taken into account, this can lead to a decline in trust and to dissatisfaction, which in turn can reduce the use of science communication. As a potential consequence, the social integration function enabling all citizens to have a common information base may not be realized. Moreover, simply having a large audience does not allow for conclusions to be drawn about either what the individuals like about the format, or whether their expectations are fulfilled. For these reasons, this article investigates both the audiences’ expectations of quality and assessments of an established and well-known science communication format: a scientific expert debate. Furthermore, the extent to which the audiences’ perceptions go hand in hand with what science communicators perceive as their tasks, what they consider as characteristics of the quality of science communication, and whether they orient their work to an imagined audience, has also not yet been researched. Therefore, this article investigates the relationship and (in)congruencies between audiences’ and science communicators’ perceptions of specific functions of science communication as well.

Adding to the academic discourse on journalistic quality, we draw on Wolling’s Theory of Subjective Quality Assessment. This approach assumes that the audience has quality expectations of media products, regarding specific characteristics of both content and style, which are related to their motives for use. The audience’s assessments of these features are the basis for their selection behavior. If individuals perceive their expectations of a specific feature as fulfilled, they are assumed to assess the format positively. The more these expectations are satisfied, the more likely the format will be used by the audience. This study adapts this media quality assessment approach to an expert debate. To operationalize potential quality criteria, Arnold’s journalistic quality dimensions were utilized and extended by specific science communication aspects. Tasks and functions ascribed to (science) journalism show strong similarities with those ascribed to science communication (e.g., information transfer, education and enlightenment, criticism and control, as well as acceptance). Therefore, this approach for evaluating the quality of science communication formats was chosen. To investigate what characteristics the audience expects from a scientific expert debate, a quantitative pre-post survey was conducted. The results are compared with findings from qualitative semi-structured interviews with science communicators who are responsible for the conceptualization of the expert debate under study. Their individual understanding of the quality of science communication as well as the goals, ideas and audience images associated with the debate format were subject matter of the interviews.

The descriptive results of the survey show that the audience expects the debate format and the debating experts to be credible, accessible, independent, neutral, and diverse. The audience also believes scientific information should be reliable and comprehensible in order to process complex, abstract scientific knowledge. These quality dimensions represent to a certain extent ‘basic prerequisites’ which enable participants to trust scientific findings and, where applicable, to take them into account when making decisions. In contrast, public engagement within an expert debate was less important for the audience. This may be due to laypeople needing to feel permitted to engage with scientific issues before beginning a dialogue. From this perspective, the attendants’ wish for factual knowledge and being informed could be understood as an ‘enabler for engagement’.

There are discrepancies between the audience’s expectations and the science communicator’s view. In contrast to the audience, they consider a high degree of topicality and social relevance to be important quality characteristics. They hope to reach those parts of the public who are disengaged with science and seem uninterested. Furthermore, the science communicators perceive dialogue and participation to be at the core of their debate format. However, they remain vague about what form of audience participation is desired. Overall, their statements reflect the tension between normative claims for science communication and their implementation in practice.

Future research may also consider other science communication formats to identify specific and overarching quality expectations of the audience. In addition, the proposal for the assessment of quality expectations developed in this article should be further elaborated on. In the future, the science communicators’ perspective could be researched more in-depth as their ideas about the success and goals of their formats significantly shape the conceptualization and development of science communication activities. All in all, further investigations that focus on quality dimensions and measurement instruments seem promising and have the potential to expand our understanding of the use and evaluation of science communication.

## Einleitung und Relevanz

Wissenschaftskommunikation trägt dazu bei, wissenschaftliches Wissen für die breite Öffentlichkeit zugänglich zu machen. Die Kommunikation über Wissenschaft erfolgt auf vielfältige Art und Weise: von klassischen Angeboten wie Diskussionsveranstaltungen und Fernsehdokumentationen über digitale Kommunikationsformen wie Online-Blogs bis hin zu Citizen-Science-Projekten (vgl. Bonfadelli et al. 2017b). Diese Ausdifferenzierung erfordert eine „weite Definition des Gegenstandsbereiches“ (Bonfadelli et al. 2017a, S. 5), sodass unter Wissenschaftskommunikation „alle Formen von auf wissenschaftliches Wissen oder wissenschaftliche Arbeit fokussierter Kommunikation, sowohl innerhalb als auch außerhalb der institutionalisierten Wissenschaft, inklusive ihrer Produktion, Inhalte, Nutzung und Wirkungen“ (Schäfer et al. 2015, S. 13) zu verstehen sind. Dazu zählen „externe und interne Wissenschaftskommunikation, darunter Wissenschaftsjournalismus, Formen der strategischen Wissenschaftskommunikation wie Wissenschafts-PR und Wissenschaftsmarketing sowie Wissenstransfer und Wissenskommunikation“ (vgl. DGPuK FG Wissenschaftskommunikation 2017), die in der Praxis teilweise miteinander verschmelzen (vgl. Schäfer 2017). Wissenschaftskommunikation nimmt eine Vermittlerfunktion zwischen Wissenschaft und Öffentlichkeit ein. Sie kann Bürger*innen Teilhabe und einen Zugang zu Themen und ein Wissen ermöglichen, das die Grundlage für individuelle wie auch politische Entscheidungen bildet, und sie zur Meinungsbildung befähigen (vgl. Schäfer et al. 2015, S. 11). Auch die gegenwärtige Covid-19-Pandemie macht vielfach die Relevanz von Wissenschaftskommunikation deutlich. Das hohe Informationsbedürfnis der Bevölkerung, aber auch Beispiele verfehlter Wissenschaftskommunikation wie der prominente Fall der Kommunikation über die von Prof. Dr. Hendrik Streeck geleitete Heinsberg-Studie (vgl. Weißkopf 2020) sowie die Veröffentlichung einer Pressemitteilung der Universität Hamburg zu einem auf ResearchGate veröffentlichten Preprint zum vermeintlichen Ursprung des Coronavirus (vgl. Weißschädel 2021) veranschaulichen eindrücklich, wie wichtig es ist, sachlich korrekte, vertrauenswürdige Inhalte zu vermitteln. Zugleich werfen sie die Frage auf, was „gute Wissenschaftskommunikation“ letztlich charakterisiert. Nicht nur die wissenschaftliche Forschung muss Qualitätsansprüchen genügen, sondern auch die Kommunikation darüber.

Eine Auseinandersetzung mit Qualitätsfragen befindet sich in der Wissenschaftskommunikation in ihren Anfängen. Standards oder Maßstäbe zur Qualitätssicherung sind noch nicht einheitlich definiert bzw. etabliert (vgl. Wormer 2017, S. 437). Sie beziehen sich bislang vermehrt auf konkrete Anwendungsfelder wie beispielsweise die „Leitlinien guter Wissenschafts-PR“ (vgl. Wissenschaft im Dialog 2016) oder die Bewertung wissenschaftsjournalistischer Berichterstattung (vgl. Rögener und Wormer 2017, 2020). Bislang wissen wir vergleichsweise wenig darüber, was das Publikum von Wissenschaftsvermittlung erwartet. Damit Wissenschaftskommunikation die Bevölkerung erreicht und ihr Zugang zu Wissenschaft sowie eine gemeinsame Informationsbasis ermöglicht, sollten die Angebote auch ihren Qualitätsansprüchen entsprechen. Eine daraufhin zielgruppengerechte Gestaltung von Wissenschaftskommunikation kann das Interesse und damit eine erhöhte Anwendung wissenschaftlicher Erkenntnisse begünstigen (vgl. Serong et al. 2019, S. 85). Erkenntnisse aus der Journalismusforschung deuten zudem darauf hin, dass es Zusammenhänge zwischen Publikumswahrnehmungen von „gutem Journalismus“ und ihrer Mediennutzung gibt (vgl. Gil de Zúñiga und Hinsley 2013, S. 935). Kritik und Unzufriedenheit hingegen können zu Vertrauensverlust und Verdrossenheit und dadurch zu Informationsvermeidung führen (vgl. Donsbach et al. 2009; Fawzi und Obermaier 2019, S. 33–37; Gurr und Metag 2021, S. 1797–1798). Dies ist aufgrund aktueller Entwicklungen wie der Verbreitung von Verschwörungsmythen sowie Missinformationen und den damit verbundenen Befürchtungen, dass demokratische Diskurse erodieren und das Vertrauen in Wissenschaft sinkt, für das Verhältnis von Wissenschaft und Öffentlichkeit von gesteigerter Bedeutung (vgl. Scheufele und Krause 2019).

Da Qualität nicht nur vom Publikum, sondern von „verschiedenen Normierungsinstanzen“ (Saxer und Kull 1981, S. 14), wie politischen Akteur*innen, rechtlichen Institutionen und Kommunikator*innen, und folglich anhand „unterschiedliche[r] Maßstäbe und Referenzsysteme (Gemeinwohl, Publikumsinteressen, ökonomischer Erfolg […])“ (Weischenberg 2006, S. 12) beurteilt wird, kann es zu Diskrepanzen zwischen den Betrachtungsperspektiven kommen. Inwiefern die Vorstellungen des Publikums mit dem einhergehen, was Kommunikator*innen bzw. Praktiker*innen als ihre Aufgaben sehen, was ihrer Ansicht nach die Qualität von Wissenschaftskommunikation kennzeichnet und ob sie sich wie Journalist*innen an ihrem (imaginierten) Publikum orientieren (vgl. Hohlfeld 2012; Meusel 2014, S. 53–56; Loosen et al. 2020, S. 1745–1748), ist ebenfalls noch nicht näher erforscht. Daher legt dieser Beitrag ergänzend Befunde zu (In‑)Kongruenzen beider Sichtweisen vor.

Als exemplarischer Untersuchungsgegenstand wird das Format „wissenschaftliche Expert*innendebatte“ herangezogen. Es ist ein bekanntes, etabliertes Informationsangebot, das konstanter Bestandteil des wissenschaftsbezogenen Informationsrepertoires ist (vgl. Wissenschaft im Dialog 2018) und den gegenwärtigen Trend zu Event- und dialogorientierten Formaten in der Wissenschaftskommunikation widerspiegelt (vgl. Fähnrich 2017, S. 167). Da es sich beim Publikum in der Regel um Befürworter*innen von Wissenschaft handelt, die an wissenschaftlichen Themen interessiert sind und Wissenschaft wertschätzen (vgl. u. a. zu „Sciencephiles“, Schäfer et al. 2018, S. 846; zu „Science Consumers“, Metag et al. 2018, S. 1087; Wicke und Taddicken 2020, S. 13), handelt es sich um eine wichtige Zielgruppe von Wissenschaftskommunikation. Daher erscheint es relevant, das Publikum einer Expert*innendebatte – und insbesondere ihre Erwartungen und Bedürfnisse – vertiefend zu betrachten. Nutzungszahlen und hohe Reichweiten lassen noch keine Rückschlüsse darauf zu, was dem Publikum an dem Format gefällt (vgl. Hasebrink 1997, S. 206), sodass es dazu befragt werden sollte. Das kann zu einem besseren Verständnis führen, wie eine Expert*innendebatte gestaltet und wie sie modifiziert werden könnte. Dazu wurde ein Qualitätskriterienkatalog zur Erhebung sowohl von Erwartungen als auch von Bewertungen im Rahmen einer quantitativen Pre-Post-Befragung des Formats entwickelt, deren Ergebnisse mit Befunden aus qualitativen Leitfadeninterviews mit Formatverantwortlichen kontrastiert werden. Die vorliegende Studie möchte damit einen Beitrag zur aktuellen Diskussion um Qualität und Evaluierung von Wissenschaftskommunikation leisten (vgl. Jensen und Gerber 2020, S. 2; Ziegler et al. 2021).

## Die Theorie der subjektiven Qualitätsauswahl

Eine Erforschung der Qualitätsvorstellungen von Wissenschaftskommunikation erfolgt orientiert an der Theorie der subjektiven Qualitätsauswahl von Wolling (vgl. Wolling 2009, 2004). Die Theorie der subjektiven Qualitätsauswahl ist ein Ansatz, um die Auswahl und Nutzung von (medialen) Angeboten anhand einer Qualitätsperspektive zu erklären. Sie wurde als Weiterentwicklung des Uses-and-Gratifications-Approach nach der Kritik an dessen „Inhaltsvergessenheit“ (Vorderer 1992, S. 32) entworfen. Entsprechend fragt Wollings Ansatz, welche inhaltlichen und gestalterischen Elemente eines Angebots für das Publikum relevant sind. Qualität wird hier verstanden als „the features of any media product (to read, view, listen to and/or interact with) that are significant in the recipient’s (or user’s) choosing to give attention to that product“ (Wolling 2009, S. 86). Jedes Angebot hat eigene Qualitäten im Sinne von Eigenschaften, anhand derer es sich von anderen unterscheiden lässt.

Wolling setzt voraus, dass 1) Menschen *Erwartungen *und Wünsche an gewisse Qualitäten von Formaten haben, die mit ihren Nutzungsmotiven zusammenhängen, und dass sie diese benennen können. Fokussiert werden sollte, welche Eigenschaften („features“) das Publikum bei einem idealen Produkt erwartet, „was sie meinen, wie etwas sein *sollte*“ (Vowe und Wolling 2004, S. 77). Es ist möglich, dass Erwartungen teilweise widersprüchlich oder nur schwer miteinander vereinbar sind. Zugleich kann eine als defizitär wahrgenommene Eigenschaft nicht dadurch ausgeglichen werden, dass die komplementäre Eigenschaft besonders ausgeprägt vorhanden ist. Die erwarteten und wahrgenommenen Qualitäten müssen bei beiden Aspekten möglichst nah beieinander liegen. Außerdem ist 2) die *Wahrnehmung* der Qualität konkreter Angebote integraler Teil des Rezeptionsprozesses, denn diese wirkt sich auf das Qualitätsurteil aus. Es handelt sich bei der Wahrnehmung um die Fähigkeit, Objekte zu beobachten und voneinander zu unterscheiden. Sie wird als ein subjektiver Konstruktionsprozess gefasst, bei dem neben den Eigenschaften des Objekts, des Formats, die Eigenschaften des Subjekts, des Publikums, relevant sind. Seine physiologischen wie psychologischen Möglichkeiten und Grenzen entscheiden mit über die Wahrnehmung (vgl. Vowe und Wolling 2004, S. 78). Das 3) *Qualitätsurteil* konstituiert sich dann anhand eines Abgleiches von Qualitätserwartungen und Qualitätswahrnehmungen (vgl. Wolling 2009, S. 87). Haben Rezipierende Erwartungen hinsichtlich spezifischer Qualitätsmerkmale und nehmen sie diese bei einem Angebot wahr, fällt ihre Bewertung positiv aus. Das wirkt sich auf Nutzungsentscheidungen aus: „Je positiver das Qualitätsurteil über ein Medienangebot ausfällt, desto höher ist die Wahrscheinlichkeit, dass dieses Angebot ausgewählt und regelmäßig genutzt wird“ (Wolling 2004, S. 174). Das Qualitätsurteil fällt sowohl positiv aus, wenn erwartete Eigenschaften wahrgenommen werden können, als auch dann, wenn Eigenschaften, die Rezipierende missbilligen, nicht vorhanden sind (vgl. Wolling 2009, S. 89).

Wollings Ansatz wird als „conceptual framework“ (Wolling 2009, S. 98) verstanden und kann zur Entwicklung von Forschungsfragen dienen. Sein recht hohes Abstraktionsniveau ermöglicht es, eine Vielzahl an Formaten einzubeziehen. Zudem erlaubt seine empirische Anwendung, die Inhalte und Eigenschaften eines Formats explizit berücksichtigt, einen hohen Grad der praktischen Anwendbarkeit der Erkenntnisse. Die Theorie der subjektiven Qualitätsauswahl wird daher in dem vorliegenden Beitrag auf das Format „Expert*innendebatte“ angewendet. Somit ergeben sich zunächst die beiden folgenden Forschungsfragen:

### FF1

Welche Qualitätserwartungen hat das Publikum an das Wissenschaftskommunikationsformat Expert*innendebatte?

### FF2

Wie bewertet das Publikum eine wissenschaftliche Expert*innendebatte?

## Qualitätserwartungen an Wissenschaftskommunikation

Die Qualitätsforschung nimmt ihren Ausgang in der Journalismusforschung. In der Kommunikationswissenschaft ist journalistische Qualität „eines der wichtigsten und zugleich komplexesten Konstrukte“ (Wellbrock und Klein 2014, S. 387). Ähnlich wie bei der Diskussion um die Qualität traditioneller Medien hat sich auch in jüngerer Zeit und insbesondere angesichts der Covid-19-Pandemie eine Debatte um die Qualität von Informationen aus der Wissenschaftskommunikation entsponnen (vgl. Wissenschaft im Dialog 2014, 2016; Wormer 2017). Damit einher gehen beispielsweise Sorgen um einen möglichen Vertrauens- und Glaubwürdigkeitsverlust der Wissenschaft (vgl. Kohring 2005; Weingart und Guenther 2016, S. 2). Im Unterschied zur Erforschung massenmedialer journalistischer Kommunikation ist der Forschungsstand zu Qualitätserwartungen und -bewertungen verschiedener Wissenschaftskommunikationsformen jedoch noch sehr überschaubar. An der Schnittstelle von Journalismus- und Wissenschaftskommunikationsforschung setzen sich Arbeiten mit der Bewertung von Umwelt- und Medizin- bzw. Gesundheitsberichterstattung auseinander (vgl. z. B. Anhäuser und Wormer 2012; Bartsch et al. 2014; Oxman et al. 1993; Rögener und Wormer 2017, 2020; Schwitzer 2008; Serong et al. 2016, 2019; Wilson et al. 2009; Wormer und Anhäuser 2014). Die bislang vorgelegten Qualitätskriterien sind allerdings inhaltlich spezifisch – sie sind themenbezogen und beziehen sich auf Print- und Online-Wissenschaftsjournalismus, sodass sie nicht uneingeschränkt zur allgemeinen Bewertung eines Wissenschaftskommunikationsformats angewendet werden können. Bislang liegt demnach noch kein einheitliches Bezugssystem für die Bestimmung der Qualität von Wissenschaftskommunikation vor: „[…] die Wissenschaftskommunikation [scheint] in 15 Jahren PUSH zunächst wenig Anstrengungen verfolgt zu haben, eigene Qualitätsmaßstäbe zu etablieren“ (Wormer 2017, S. 437). Es mangelt sowohl an weiteren theoretischen Ausarbeitungen als auch an zugehörigen empirischen Messinstrumenten, mit denen verschiedene Formen untersucht werden könnten. Wormer schlägt vor, etablierte Qualitätskriterien aus dem (Wissenschafts‑)Journalismus heranzuziehen, was ihm „angesichts des konstatierten Mangels an etablierten Kriterien zur Sicherung der publizistischen Qualität solcher Produkte […] plausibel“ (Wormer 2017, S. 441) erscheint. Aufgaben und Funktionen wie Informationstransfer, Bildung und Aufklärung, Kritik und Kontrolle sowie Akzeptanz werden sowohl dem (Wissenschafts‑)Journalismus (vgl. Kohring 2012, S. 132–135) als auch Wissenschaftskommunikation zugeschrieben (vgl. z. B. acatech – Deutsche Akademie der Technikwissenschaften 2017; Gantenberg 2018; Weitze und Heckl 2016). So plädieren Wormer und Anhäuser (2014, S. 35) dafür, anstatt die Qualität von (Wissenschafts‑)Journalismus und Wissenschaftskommunikation nach getrennten Kriterien zu betrachten, „akteursübergreifende Dimensionen für die Qualitätsbewertung […] zu erarbeiten“. Zudem gelte es verstärkt „angesichts der zunehmenden Konvergenz von Wissenschaftsjournalismus und Wissenschafts-PR […], auch die Perspektive des Endnutzers einzunehmen“ (Serong et al. 2016, S. 117). Diese wurde bislang überwiegend anhand von Bewertungen von Wissenschaftssendungen in qualitativen Forschungsdesigns erforscht (vgl. de Cheveigné und Véron 1996; Cooper et al. 2001; Dehm 2008; Maier et al. 2016; Milde 2009; Milde und Barkela 2016; Taddicken und Wicke 2019; Wicke und Taddicken 2021). Dabei wurde u. a. ermittelt, dass dem Publikum eine verständliche Erklärung wissenschaftlicher Inhalte, die oft abstrakt, komplex und damit schwer nachvollziehbar sind, besonders wichtig ist. Darunter fällt, dass die Verwendung von Fachbegriffen und Wissenschaftsjargon vermieden und wissenschaftliche Erkenntnisse kontextualisiert werden sollten (vgl. Bullock et al. 2019, S. 850; Taddicken et al. 2020, S. 61–62; Wicke und Taddicken 2021, S. 55–57). Außerdem wünscht sich das Publikum Informationen zu wissenschaftlichen Forschungsprozessen und -methoden und möchte über Unsicherheiten und Widersprüchlichkeiten wissenschaftlicher Erkenntnisse informiert werden (vgl. Maier et al. 2016, S. 255–256; Milde und Barkela 2016, S. 200–202). Gute Wissenschaftskommunikation beinhaltet nach Ansicht des Publikums außerdem, dass die Vielfalt von (wissenschaftlichen) Perspektiven und Disziplinen, die an der Erforschung eines Themas beteiligt sind, dargestellt und die Alltagsrelevanz aufgezeigt, d. h. eine „praktische Anwendung“ der wissenschaftlichen Erkenntnisse ermöglicht wird. Positiv wahrgenommen wird zudem, wenn die Inhalte über einen gewissen Unterhaltungs- und Neuigkeitswert verfügen (vgl. Taddicken und Wicke 2019, S. 161–163; Wicke und Taddicken 2021, S. 56). Inwiefern sich die Erwartungen hinsichtlich ihrer Wichtigkeit voneinander unterscheiden, wird anhand folgender Forschungsfrage untersucht:

### FF3

Welche Relevanz schreibt das Publikum einzelnen Qualitätsdimensionen zu?

Ein solches Ranking ermöglicht die Vergleichbarkeit der Dimensionen untereinander und bietet konkrete Ansatzpunkte, worauf das Publikum Wert legt, sowie Orientierungshilfen, inwiefern Praktiker*innen das Format Expert*innendebatte optimieren können. Zudem liegt die Vermutung nahe, dass von den als besonders wichtig empfundenen Dimensionen ein größerer Einfluss auf die Urteilsbildung ausgehen könnte.

Auch ein Vergleich der Publikumserwartungen mit denen der Kommunikationsverantwortlichen wurde noch nicht vorgenommen. Bisherige Forschung zu Wissenschaftskommunikator*innen bezieht sich in der Regel auf kommunizierende Wissenschaftler*innen und von ihnen wahrgenommene Einflüsse auf ihr Public Engagement-bezogenes Verhalten, darunter Motivationen und Hindernisse auf institutioneller und individueller Ebene (vgl. z. B. Cerrato et al. 2018; Ho et al. 2020; Baram-Tsabari und Lewenstein 2017; Besley et al. 2020). Was aus Sicht der Formatentwickler*innen erfolgreiche Wissenschaftskommunikation kennzeichnet und welche Ziele sie verfolgen, bedarf weiterer Untersuchungen. Aus der Journalismusforschung ist bekannt, dass sich Einschätzungen von Bürger*innen und Journalist*innen in Bezug auf Rollenwahrnehmungen sowie Aufgaben und Leistungen unterscheiden können (vgl. Loosen et al. 2020; Vos et al. 2019; Willnat et al. 2019). Daher fragt der vorliegende Beitrag:

### FF4

Welche (In‑)Kongruenzen lassen sich feststellen zwischen dem, was das Publikum und was Kommunikator*innen von Wissenschaftskommunikation erwarten?

## Untersuchungsgegenstand, methodisches Vorgehen und Operationalisierung

Bei dem fokussierten Format handelt es sich um eine öffentliche, kostenfreie 90-minütige Expert*innendebatte, die als Veranstaltungsreihe in verschiedenen deutschen Städten stattfindet. Ihr Ziel ist es, wissenschaftliche Erkenntnisse und Perspektiven verständlich zu vermitteln, faktenbasierte Debatten zu gesellschaftlich relevanten Themen anzustoßen und den Austausch zwischen Wissenschaft und Öffentlichkeit zu fördern. An der von zwei bekannten Wissenschaftsjournalist*innen moderierten Diskussion nehmen üblicherweise drei Expert*innen aus unterschiedlichen wissenschaftlichen Disziplinen teil. Angesichts der steigenden Relevanz von Onlinekommunikation wird jede Debatte zudem live über einen YouTube-Kanal und die Homepage eines Medienpartners gestreamt. Das Publikum kann sich vor Ort und über soziale Medien an die Expert*innen wenden. Entsprechend handelt es sich nicht um ausschließlich non-mediale Präsenzkommunikation, sondern um eine Art medial inszeniertes Hybrid-Format.

Das Format wurde anhand eines Mixed-Method-Designs untersucht. Um die Publikumsperspektive zu beforschen, bot sich orientiert an den Prämissen der Theorie der subjektiven Qualitätsauswahl (vgl. Wolling 2009) eine quantitative Pre-Post-Befragung an, um neben einem Qualitätsurteil vorab Erwartungen zu erheben, sodass ein Abgleich und Rückschlüsse auf die leitenden Qualitätsmaßstäbe möglich sind. Die Perspektive der Formatverantwortlichen wurde anhand leitfadengestützter Expert*inneninterviews untersucht, um ihr Selbstverständnis und Hintergründe zur Konzeption zu explorieren.

### Pre-Post-Befragung des Publikums

Die Datenerhebung fand im Rahmen einer Veranstaltung zum aktuellen und kontroversen sozial-, wirtschafts- und ingenieurwissenschaftlichen Thema „Wohnungsmarkt“ statt. Auf dem Podium diskutierten drei Professor*innen für Geographische Stadtforschung, Infrastruktur- und Immobilienmanagement sowie Stadt- und Regionalsoziologie u. a. über Mietpreisbremse, soziale Segregation, Gentrifizierung, nachhaltigen Wohnungsbau sowie Entwicklung, Finanzierung und Betrieb von Immobilienprojekten. Die Teilnehmenden füllten direkt vor und unmittelbar nach der Veranstaltung Papierfragebögen aus.

#### Messvariablen

Zur Entwicklung eines Instruments, mit dem sich die Qualität von Wissenschaftskommunikation bzw. einer wissenschaftlichen Expert*innendebatte aus Publikumsperspektive messen lässt, wurde auf das integrative Qualitätskonzept von Arnold (2009) zurückgegriffen, das als „Meilenstein“ (Wyss 2011) der journalistischen Qualitätsforschung bezeichnet wird. Arnold (2009) leitet die jeweiligen Zusammenhänge theoretisch her und verortet die Qualitätskriterien auf drei theoretischen Ebenen: Auf der (1) funktional-systemorientierten Ebene identifiziert er als Qualitätsdimensionen Vielfalt, Aktualität, Relevanz, Glaubwürdigkeit, Unabhängigkeit, Recherche, Kritik und Zugänglichkeit/Verständlichkeit; auf der (2) normativ-demokratieorientierten Ebene verortet er Ausgewogenheit, Neutralität/Trennung von Nachricht und Meinung sowie Achtung der Persönlichkeit und auf der (3) nutzerbezogen-handlungsorientierten Ebene die beiden Kriterien Anwendbarkeit und Unterhaltsamkeit.^1^ Arnold bestimmt die Qualität von Journalismus demnach anhand grundlegender Funktionen und Aufgaben, Rollenselbstverständnis und Berufsnormen sowie gesellschaftlichen (Grund‑)Werten, medienrechtlichen Bestimmungen und Publikumsinteressen. Die Qualitätsdimensionen, die aus dem Forschungsstand zu Publikumserwartungen an Wissenschaftskommunikation abgeleitet werden können (Kap. 3), finden sich hier bereits wieder: *Verständlichkeit, Ausgewogenheit, Vielfalt, Anwendbarkeit* und *Unterhaltsamkeit*. Zusätzlich wurden fünf weitere Dimensionen ergänzt, um Wissenschaftskommunikations- bzw. formatspezifische Aspekte zu berücksichtigen (vgl. zur zusätzlichen Kriterienentwicklung Göpfert 1993, S. 106). Darunter fallen *Bildung, Aufklärung und Information* sowie *Legitimation und Akzeptanz von Wissenschaft* als zentrale Aufgaben von Wissenschaftskommunikation (vgl. z. B. acatech – Deutsche Akademie der Technikwissenschaften 2017; Burns et al. 2003; Gantenberg 2018; Weitze und Heckl 2016). Die Bevölkerung soll informiert und aufgeklärt, ihr Verständnis von und für Wissenschaft gefördert werden. Da Wissenschaft u. a. von gesellschaftlichen Ressourcen und dem Vertrauen der Bevölkerung abhängig ist, muss sie sich auch ihre öffentliche Legimitation sichern (vgl. Weingart 2011, S. 45–46). Eine zunehmende Orientierung am Public Engagement-Paradigma in der Vermittlung hat die Bedeutung der Interaktion zwischen Wissenschaft und Öffentlichkeit wachsen lassen und zahlreiche Formate hervorgebracht, sodass *Dialog und Partizipation* eine weitere Dimension darstellen (vgl. Bubela et al. 2009; Fähnrich 2017; van der Sanden und Meijman 2008). Außerdem wurde *Emotionen* aufgenommen, da Wissenschafts- und Umweltthemen nicht nur sachlich und evidenzbasiert vermittelt werden, sondern vielfach eine Emotionalisierung zu beobachten ist (vgl. Huber und Aichberger 2020; Lidskog et al. 2020; Taddicken und Reif 2020). In welchem Verhältnis sich Emotionen und Informationen beziehungsweise Wissen zueinander befinden, inwieweit Emotionen in der Kommunikation notwendig sind und wie diese vom Publikum wahrgenommen werden, wird beispielsweise bereits im Kontext der Klimawandeldarstellung intensiv untersucht (vgl. Chapman et al. 2017; Nabi et al. 2018; O’Neill und Nicholson-Cole 2009). Zuletzt wurden *Rahmenbedingungen* ergänzt, um veranstaltungsorganisatorische Aspekte zu berücksichtigen, ähnlich den Kriterien, die die ästhetische Gestaltung eines Medienproduktes erfassen (vgl. Dahinden et al. 2004; Göpfert 1993; Tab. 1).

**Table 1:** 

Ebene	Qualitätsdimension
*1) Funktional-systemorientierte Ebene*	Vielfalt Aktualität Relevanz Glaubwürdigkeit Unabhängigkeit Recherche Kritik Zugänglichkeit/Verständlichkeit
*2) Normativ-demokratieorientierte Ebene*	Ausgewogenheit Neutralität Achtung der Persönlichkeit
*3) Nutzerbezogen-handlungsorientierte Ebene*	Anwendbarkeit Unterhaltsamkeit
*4) Wissenschaftskommunikations- bzw. formatspezifische Ebene*	Bildung, Aufklärung und Information Legitimation und Akzeptanz Dialog und Partizipation Emotionen Rahmenbedingungen

Da konkrete Eigenschaften des Formats bei der Qualitätsbestimmung relevant sind (vgl. Wolling 2009, S. 86), wurden alle 18 Dimensionen dahingehend betrachtet, inwiefern sie sich auf die drei charakteristischen Bezugsobjekte a) das Format, b) die wissenschaftlichen Expert*innen sowie c) das inhaltliche Thema beziehen lassen, und entsprechend Items gebildet (Anhang, Tab. 4). Insgesamt setzt sich der Pre-Fragebogen aus 43 Items, der Post-Fragebogen aus 69 Items zusammen, deren Wichtigkeit auf einer fünfstufigen Likert-Skala beurteilt wurde. Die Skalen des Pre-Fragebogens sowie des Post-Fragebogens wiesen eine hohe interne Konsistenz auf (Cronbachs α = 0,92; 0,89).

Neben den operationalisierten Qualitätsdimensionen wurden sechs offene, explorierende Fragen integriert, die um Rückmeldung baten, inwiefern es beispielsweise gelang, das Publikum einzubeziehen und wodurch sich eine sehr gute Expert*innendebatte auszeichnet. Weitere Fragen erfassten u. a. das wissenschaftsbezogene Informationsverhalten, Einstellungen gegenüber Wissenschaft sowie das Gesamturteil und die Absicht, das Format erneut zu besuchen bzw. weiterzuempfehlen.

#### Stichprobe

Die lokale Rekrutierung der Teilnehmenden erfolgte über Flyer sowie Online-Aufrufe. Sie erhielten für ihre ca. zweistündige Studienteilnahme eine Aufwandsentschädigung in Höhe von 20 €. Das somit auf Selbstselektion basierende, nicht-bevölkerungsrepräsentative Sample setzt sich aus 40 Proband*innen zusammen (Tab. 2). Das Durchschnittsalter liegt bei 31,8 Jahren (*SD* = 11,68), die Altersspanne bei 21 bis 64 Jahren. Die größte vertretene Bildungsgruppe sind Personen mit Hochschulabschluss, die das typische Publikum einer wissenschaftlichen Expert*innendebatte widerspiegeln (vgl. Wicke und Taddicken 2020, S. 13).

**Table 2:** 

Stichprobe	(*n* = 40)
*Geschlecht *[%]	
Männlich	55 %
Weiblich	45 %
*Alter* (M *[SD]*)	31,82 *(11,68)*
*Formale Bildung *[%]	
Hochschulabschluss	45 %
Abitur (Hochschulreife)	40 %
Fachhochschulreife	12,5 %
Mittlere Reife, Realschulabschluss	2,5 %
*Interesse an Wissenschaft*^a^ (M *[SD]*)	
An wissenschaftlichen Themen	4,40 *(0,87)*
An der Arbeitsweise von Wissenschaftler*innen	3,74 *(1,04)*
Am Wissenschaftssystem im Allgemeinen	3,63 *(1,10)*

^a^Fünfstufige Skala von 1 = „überhaupt kein Interesse“ bis 5 = „voll und ganz interessiert“

### Leitfadengestützte Expert*inneninterviews mit Verantwortlichen des Wissenschaftskommunikationsformats

Für die Kommunikator*innenperspektive stellen ca. 60minütige Expert*inneninterviews mit fünf Projektverantwortlichen des Debattenformats die empirische Grundlage dar. Unter ihnen sind Wissenschaftsjournalist*innen und (Hochschul‑)Kommunikator*innen in leitenden Funktionen. Die Gespräche wurden während der Konzeptualisierungsphase geführt, bevor das Format realisiert wurde. Um den Sinn und die Bedeutung zu erfassen, die die Kommunikator*innen Wissenschaftskommunikation sowie ihrem Format zuschreiben, umfasste der in einem Pretest validierte Leitfaden (vgl. Loosen 2016) folgende wesentliche Themenbereiche: das institutionelle und individuelle Qualitätsverständnis von Wissenschaftskommunikation sowie die mit dem Debattenformat und seiner Konzeption verbundenen Ziele, Vorstellungen und Publikumsbilder (Anhang, Tab. 5). Nachdem eine theoretische Sättigung erreicht und keine neuen analytischen Inhalte durch die Interviews generiert werden konnten (vgl. Nelson 2017), erfolgte die Auswertung der transkribierten Inhalte mithilfe einer qualitativen, strukturierenden und zusammenfassenden Inhaltsanalyse, angelehnt an Mayring (vgl. Mayring und Fenzl 2019). Im konsensualen und iterativen Kodierprozess interessierten insbesondere Funktionen und Qualitätsansprüche.

## Ergebnisse

### Die Qualität(en) des Formats Expert*innendebatte aus Publikumsperspektive

**Figure 1:**
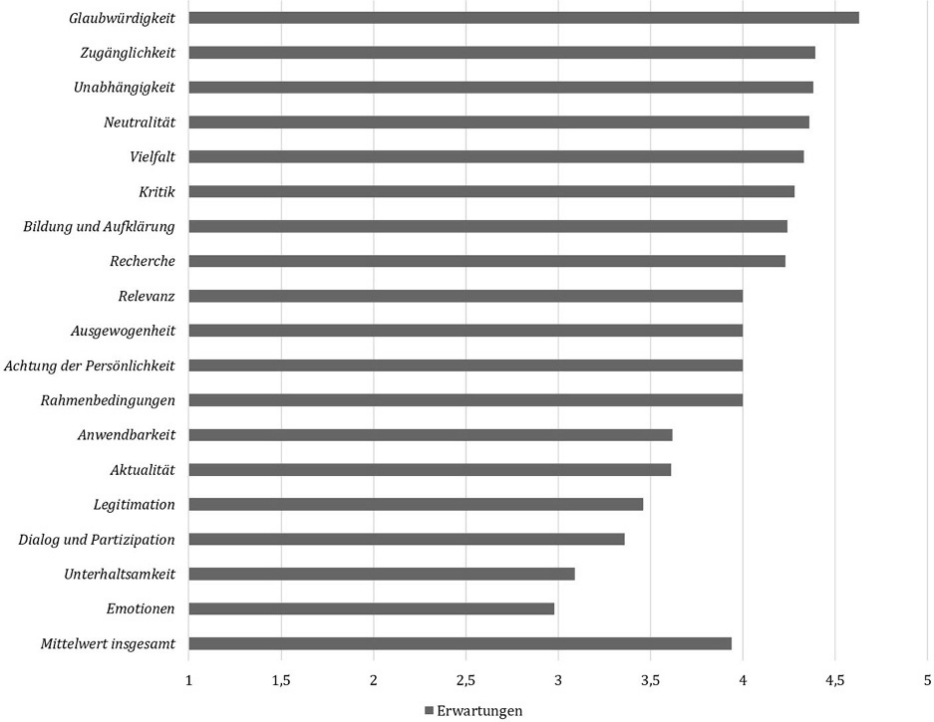

Das Publikum erwartet vom Format Expert*innendebatte (FF1, FF3) vor allem glaubwürdige und faktenbasierte Inhalte (Abb. 1, Tab. 3*Erwartung* sowie Tab. 4*Pre-Items*). Die Teilnehmenden legen Wert darauf, dass eine hohe Verständlichkeit herrscht, und erwarten, dass die beteiligten Expert*innen unabhängig wie auch objektiv sind. Sie möchten das debattierte Thema aus unterschiedlichen Blickwinkeln kennenlernen und wünschen sich, dass die Expert*innen kritisch sowie inhaltlich gut vorbereitet sind. Wichtig ist ihnen ebenfalls, dass sie als Teilnehmende etwas über das Thema dazulernen können. Demensprechend sollte ausgewogen und umfassend debattiert werden. Das Publikum erwartet hierbei auch die Thematisierung der Unsicherheit wissenschaftlicher Erkenntnisse.

**Table 3:** 

Dimension^a^	Erwartung (Pre) (M *[SD]*)	α	Bewertung (Post) (M *[SD]*)	α	df	t	Differenz (Bewertung-Erwartung) (M *[SD]*)
*Vielfalt*	4,33 *(0,60)*	0,531	3,49 *(0,69)*	0,638	39	7,124***	−0,84 *(0,75)*
*Aktualität*	3,61 *(1,14)*	0,838	4,51 *(0,66)*	0,815	39	−4,684***	0,90 *(1,22)*
*Relevanz*	4,00 *(0,87)*	0,533	3,84 *(0,60)*	−0,270	39	1,220	−0,16 *(0,84)*
*Glaubwürdigkeit*	4,63 *(0,59)*	0,783	4,22 *(0,51)*	0,694	39	4,400***	−0,41 *(0,59)*
*Unabhängigkeit*	4,38 *(0,77)*	0,545	3,96 *(0,67)*	0,607	39	3,056**	−0,41 *(0,86)*
*Recherche*	4,23 *(0,62)*	0,343	4,31 *(0,51)*	0,526	39	−0,763	0,08 *(0,67)*
*Kritik*	4,28 *(0,75)*	–	3,70 *(0,70)*	0,425	39	4,101***	−0,58 *(0,87)*
*Zugänglichkeit*	4,39 *(0,64)*	0,792	3,87 *(0,49)*	0,431	39	5,070***	−0,53 *(0,66)*
*Ausgewogenheit*	4,00 *(0,62)*	0,633	3,31 *(0,60)*	0,641	39	6,163***	−0,70 *(0,72)*
*Neutralität*	4,36 *(0,87)*	–	3,86 *(0,49)*	−0,006	38	3,278**	−0,50 *(0,96)*
*Achtung der Persönlichkeit*	4,00 *(0,99)*	–	4,52 *(0,49)*	0,666	35	−3,212**	0,48 *(0,90)*
*Anwendbarkeit*	3,62 *(0,63)*	0,581	3,12 *(0,62)*	0,626	39	4,210***	−0,50 *(0,75)*
*Unterhaltsamkeit*	3,09 *(1,07)*	0,849	2,44 *(0,82)*	0,740	39	2,931**	−0,65 *(1,40)*
*Bildung und Aufklärung*	4,24 *(0,62)*	0,744	3,66 *(0,59)*	0,688	39	5,335***	−0,58 *(0,69)*
*Legitimation*	3,46 *(1,12)*	–	2,54 *(1,21)*	–	37	3,666**	−0,92 *(1,55)*
*Dialog und Partizipation*	3,36 *(0,77)*	0,558	3,57 *(0,66)*	0,684	39	−1,334	0,21 *(0,98)*
*Emotionen*	2,98 *(1,17)*	0,838	2,60 *(0,73)*	0,689	39	1,690	−0,37 *(1,40)*
*Rahmenbedingungen*	4,00 *(0,91)*	–	4,08 *(0,97)*	–	39	−0,476	0,08 *(1,00)*
*Mittelwert insgesamt*	3,94 *(0,44)*	–	3,65 *(0,37)*	–	–	–	−0,31 *(0,41)*

***p < 0,001; **p < 0,01

^a^Aus allen der Dimension zugehörigen Items wurden Mittelwertindizes berechnet

Dass eine Expert*innendebatte emotional und unterhaltsam ist, erwarten die Teilnehmenden hingegen eher weniger. Ebenso haben sie vergleichsweise geringe Erwartungen hinsichtlich eines Public Engagements. Ihrer Ansicht nach muss das Publikum nicht unbedingt in die Debatte einbezogen werden und Fragen an Expert*innen stellen können. Für seine Einbindung spricht den Teilnehmenden zufolge, dass das Publikum Impulse geben und andere Blickwinkel aufzeigen könne. Dagegen spreche, dass es mit seinem geringen Wissensstand inhaltlich nur wenig beitragen könne. Zudem müssten gut vorbereitete Debatten ihr Publikum nicht unbedingt mit einbeziehen, da relevante Aspekte bzw. mögliche Publikumsfragen vorab recherchiert worden sein sollten. Wichtig ist den Teilnehmenden jedoch, dass die Expert*innen prinzipiell dialogbereit sind. Weiterhin nachrangig sehen sie, dass die Bedeutung von Wissenschaft für die Gesellschaft im Rahmen einer Veranstaltung deutlich werden sollte. Sie erwarten auch weniger, dass das debattierte Thema von Aktualität gekennzeichnet ist und die Expert*innen ihnen konkrete Handlungsempfehlungen geben.

**Figure 2:**
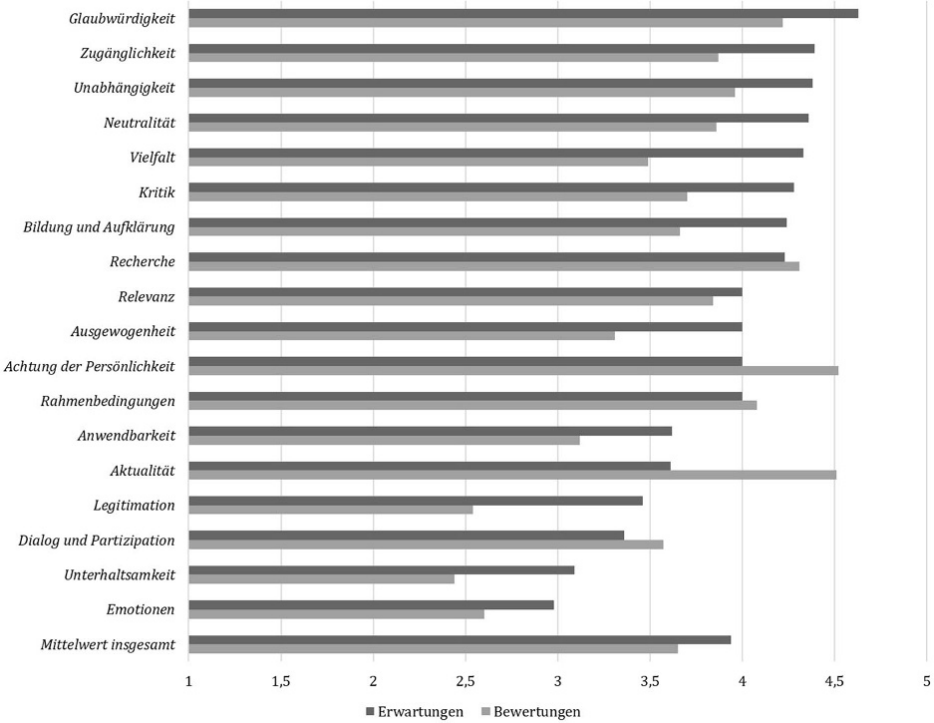

In ihrer Bewertung (FF2) geben die Teilnehmenden an, dass es den Expert*innen besonders gut gelungen ist, einen respektvollen Umgang miteinander gewahrt zu haben (Abb. 2, Tab. 3*Bewertung* sowie Tab. 4*Post-Items*). Die Befragten beurteilen zudem das diskutierte Thema Wohnungsmarkt als sehr aktuell und die Expert*innen als inhaltlich gut vorbereitet. In der offenen Beurteilung werden ihre verständliche Ausdrucksweise und ihr fundiertes Wissen hervorgehoben. Neben sachlichen Faktoren äußern die Befragten, dass die Expert*innen sympathisch und höflich waren sowie eine „nette Ausstrahlung“ hatten, d. h. in ihre Bewertung sind womöglich auch Aspekte ohne direkten Kommunikations- und Inhaltsbezug eingeflossen.

Obwohl die Publikumserwartungen über alle Dimensionen hinweg betrachtet an das Format Expert*innendebatte gut, wenn auch nicht gänzlich von der Veranstaltung erfüllt wurden (vgl. Tab. 3*Differenz*: *M* = −0,31 [*SD* = 0,41]), wird sie insgesamt nur als mittelmäßig gelungen befunden. Der Mittelwert aller Bewertungen liegt bei *M* = 3,65 (*SD* = 0,37), das abgefragte Gesamturteil fällt leicht negativer aus (*M* = 3,48; *SD* = 0,75). Das kann daran liegen, dass insbesondere die für die Teilnehmenden sehr relevanten Erwartungen an Glaubwürdigkeit, Zugänglichkeit, Unabhängigkeit, Neutralität und Vielfalt nicht erfüllt wurden (Abb. 2). Entsprechend der Theorie der subjektiven Qualitätsauswahl (vgl. Wolling 2004, 2009) kann das nicht damit ausgeglichen werden, dass ihre Erwartungen bei weniger relevanten Dimensionen wie Aktualität erfüllt bzw. deutlich übertroffen wurden. Die erwarteten und wahrgenommenen Qualitäten müssen möglichst nah beieinander liegen, um zu einem positiven Qualitätsurteil zu führen. Die Abfrage der Erwartungen bietet daher einen Erklärungsansatz für das Gesamturteil. Eine reine Abfrage der Bewertungen ließe keine Erkenntnisse zur Einordnung der Relevanz sowie zur Notwendigkeit der Veränderung der jeweiligen Dimension zu.

Die offene Frage, wie gut den Teilnehmenden die Expert*innendebatte insgesamt gefallen hat, ermöglicht weitere Einblicke: Positiv anerkannt werden die Strukturierung der Diskussion und ausgeglichene Redezeiten sowie die Differenziertheit der Debatte, u. a. im Hinblick auf die Perspektiven der Expert*innen und „Lösungsansätze“. Dass es zu keiner kontroversen Diskussion gekommen sei und zu viel Konsens unter den Expert*innen geherrscht habe, wird hingegen bemängelt. Die Expert*innen seien wenig aufeinander eingegangen, und die Debatte habe eher einen Vorlesungscharakter gehabt. Mehrfach wird erwähnt, dass ein politische*r Vertreter*in gefehlt habe. Negativ wahrgenommen wird auch, dass es zu warm im Raum gewesen sei. Als Verbesserungsvorschläge werden „mehr Leidenschaft“ und eine „kritischere und informativere“ Diskussion genannt. Außerdem seien eine visuelle Unterstützung, eine Verbesserung der Tonqualität sowie Sitzkissen wünschenswert. Zu der abschließenden Frage, was eine sehr gute Expert*innendebatte charakterisiere, finden sich „Belege durch Studien und Argumente“, eine „fundierte Abwägung von Erkenntnissen, Zusammenhängen und politischer Wille/Möglichkeit“ sowie „Interaktion zwischen den Gesprächsteilnehmern“ unter den Antworten. „Interaktion und Leidenschaft gleichermaßen wie wissenschaftlicher Input“ oder „verständlich, informierend, faktenbasiert und leidenschaftlich“ sind Qualitätskriterien, die Teilnehmende formulieren.

### (In‑)Kongruenzen der Qualitätsvorstellungen

Die Projektverantwortlichen sind sich einig, dass es „eine der Hauptanforderungen an Wissenschaft und Wissenschaftskommunikation [ist], antiwissenschaftlichen Tendenzen“ (K5) entgegenzuwirken. Zudem sehen sie es als ihre Aufgabe, Meinungsbildungs- und Entscheidungskompetenzen von Bürger*innen zu stärken und sie dabei zu unterstützen, vertrauenswürdiges Wissen einordnen zu können. Wissenschaftskommunikation lege dazu das „Fundament“ (K1). Wissen und Wesen der Wissenschaft sollen dargestellt, d. h. Prozesse und Methoden erklärt werden: „[Gute Wissenschaftskommunikation] vermittelt, wie Wissenschaft arbeitet, und warum sie manchmal komplex ist, warum sie nicht immer einfache Antworten gibt und was die Leute treibt, die Wissenschaft betreiben“ (K3). Die Verantwortlichen sehen Legitimation als weitere zentrale Funktion, da Wissenschaft der Gesellschaft rechenschafts- und erklärungsbedürftig sei. Reputationskommunikation ist für sie allerdings eine schlechte Form von Wissenschaftskommunikation.

Worauf sie bei ihrem Debattenformat Wert legen, unterscheidet sich teilweise von den Publikumserwartungen (FF4). Ihnen ist im Gegensatz zum Publikum besonders wichtig, dass Themen von hoher Aktualität („oberstes Kriterium“ [K5]) und gesellschaftlicher Relevanz diskutiert werden. Die diskutierten Inhalte sollen einen Alltagsbezug aufweisen, damit sie als Argumentationsgrundlage in persönlichen Gesprächen eingebracht werden können. Sie erhoffen sich dadurch „Leute [zu] erreichen, die grundsätzlich gesellschaftspolitisch interessiert sind, aber vielleicht nicht unbedingt wissenschaftsinteressiert“ (K2). Zugleich gehen sie aber davon aus, dass es nur schwer gelingen werde, Wissenschaftsdesinteressierte und -skeptiker*innen anzusprechen. Ein*e Kommunikator*in vermutet, dass sogar die Debattenteilnehmenden das Format nicht aufgrund eines Informationsbedürfnisses besuchen: „Aber ich glaube, die meisten Leute gucken sich Debatten an, weil es einfach so wie ein Tennisspiel ist: Es ist spannend, wer gewinnt“ (K3). Ähnlich wie von Journalist*innen wird der Publikumswunsch nach Information unterschätzt (vgl. Scholl et al. 2014, S. 26). Die Projektverantwortlichen sehen zudem Dialog und Partizipation im Kern ihres Formats, erachten jedoch das Beteiligungsbedürfnis als gering und bleiben vage, was deren konkrete Umsetzung bedeute und welche Form der Publikumsbeteiligung gewollt sei. Es ist von „Zuschaueraktivierung“ (K4), „eigene Positionen einbringen“ (K3) und „Reaktionen einholen“ (K1) die Rede. Zugleich stellt sich ein*e Kommunikator*in vor, dass das Format „Leuten die Augen öffnet, dass man Wissen an Leute tragen kann, wo jeder persönlich so einen Aha-Effekt erlebt und denkt, ‚das wusste ich gar nicht‘“ (K1).

Die dem Publikum bedeutsamen Dimensionen Glaubwürdigkeit, Unabhängigkeit und Neutralität thematisieren die Formatverantwortlichen ebenfalls. Wissenschaftskommunikation soll möglichst authentisch sein und die Leidenschaft der Forscher*innen transportieren: „Der Wissenschaftler sollte als Gesicht, als Person sichtbar sein. Das ist für mich Wissenschaftskommunikation“ (K2). Sie vermuten, das Publikum wünsche sich, dass Expert*innen integer sind und ehrlich kommunizieren, stellen allerdings in Frage, inwiefern Wissenschaftler*innen objektiv und frei von Interessenskonflikten sein können. Sie erwarten zudem, dass kommunizierte Inhalte vollständig, wahrhaftig, „überprüfbar und transparent“ (K3) sind. Ähnlich wie den Teilnehmenden ist den Verantwortlichen wichtig, dass die Vielfalt wissenschaftlicher Disziplinen und Perspektiven deutlich wird: „Ich glaube, wenn das Publikum nach der Debatte die Pluralität von Wissenschaft begreift, dann haben wir schon ganz schön viel erreicht“ (K2). Als Kommunikationsaufgabe sehen sie außerdem, dass Forschung nachvollziehbar wird. Hierbei erwarten sie wie das Publikum eine hohe Verständlichkeit. Die Kommunikation soll „möglichst fundiert und korrekt, aber trotzdem auch sehr kurz und prägnant“ (K1) sein und sich „relativ einfacher Sprache“ (K3) bedienen, um sich von innerwissenschaftlichen Diskursen abzugrenzen. Weitere Qualitätsmerkmale sind ihrer Meinung nach, dass gute Wissenschaftskommunikation Faszination vermittelt und Wissenschaft spannend und anschaulich darstellt. Sie ist außerdem strategisch geplant, d. h. Ziele, Zielgruppen und Maßnahmen sind durchdacht sowie aufeinander abgestimmt. Dies kann allerdings mit Interessen von Fördernden kollidieren. Mehrfach weisen die Kommunikator*innen darauf hin, dass sie konform mit den „Leitlinien zur guten Wissenschafts-PR“ (vgl. Wissenschaft im Dialog 2016) agieren möchten. Nicht zuletzt verstehen sie gute Wissenschaftskommunikation als progressiv, dynamisch und bereit, sich weiterzuentwickeln.

## Diskussion, Reflexion und Ausblick

Das Ziel des vorliegenden Beitrags ist es, eine neue Perspektive auf die bisherige Erforschung von Wissenschaftskommunikation zu eröffnen, indem er eine Qualitätsbestimmung von Formaten vorschlägt. Auf theoretischer Ebene angebunden an die Theorie der subjektiven Qualitätsauswahl (vgl. Wolling 2009) werden anhand der aus der journalistischen Qualitätsforschung transferierten und erweiterten Qualitätsdimensionen von Arnold (2009) die Eigenschaften und Inhalte einer wissenschaftlichen Expert*innendebatte aus Publikumssicht betrachtet. Dabei wird das im Anschluss an die Rezeption erfragte Urteil durch die Erhebung von Qualitätserwartungen kontextualisiert. Die so ermittelte Publikumsperspektive wird empirisch ergänzt um die Frage, was sich die verantwortlichen Kommunikator*innen von dem Format erhoffen.

Die Befunde zeigen, dass das Publikum von einer Expert*innendebatte besonders Glaubwürdigkeit, Zugänglichkeit/Verständlichkeit, Unabhängigkeit, Neutralität und Vielfalt erwartet, was zentrale Werte der Wissenschaft widerspiegelt und ihre Schutzwürdigkeit unterstreicht. Diese Dimensionen stellen gewissermaßen Grundvoraussetzungen dar, damit Teilnehmende den vermittelten Inhalten vertrauen und sie gegebenenfalls in ihrem Verhalten berücksichtigen können. Im Kontext einer Expert*innendebatte spielt Verständlichkeit eine hervorgehobene Rolle, da systematische Wissensdivergenzen zwischen wissenschaftlichen Expert*innen und Publikum (vgl. Bromme und Jucks 2018) und ein u. a. durch Fachbegriffe, Fremdwörter und Abstrakta gekennzeichneter Wissenschaftsjargon Verstehensprozesse erschweren können (vgl. Bullock et al. 2019). Bisherige Forschung zeigt, dass es zudem Zusammenhänge zwischen Sprachstil, Glaubwürdigkeit und Neutralität gibt. Beispielsweise wurden im Kontext wissenschaftlicher Gesundheitsinformationen neutral formulierte Empfehlungen glaubhafter wahrgenommen als solche, die viele positive Adjektive enthielten (vgl. König und Jucks 2020, S. 5). Die Teilnehmenden erwarten des Weiteren, dass in einer Expert*innendebatte ausgewogen und vielfältig debattiert wird, worunter sie auch Einblicke in methodische Prozesse und das Aufgreifen der Unsicherheit wissenschaftlicher Erkenntnisse verstehen. Das kann die Wissenschaftskommunikationspraxis ermutigen, nicht nur auf Erkenntnisse abzustellen, sondern auch wissenschaftliche Prozesse und Arbeitsweisen intensiver zu vermitteln, was wiederum die „scientific literacy“ (vgl. Miller 1983) stärken könnte.

Obwohl Partizipation als „gold standard“ (Felt und Fochler 2008, S. 489) zu gelten scheint und der Fokus auf Public Engagement zu einer regelrechten „participation explosion“ (Einsiedel 2008, S. 173) führte, findet sich die partizipativen Formen zugeschriebene Wichtigkeit in der Perspektive der Teilnehmenden nicht wieder. Ihr Hauptaugenmerk liegt nicht auf der Publikumseinbindung, sondern ihnen ist wichtig, dass sie etwas über das debattierte Thema dazulernen; ein Befund, der von weiteren Untersuchungen zu Expert*innendebatten- und Science Festival-Besucher*innen bestätigt wird (vgl. z. B. Fogg-Rogers et al. 2015, S. 436). Der Wunsch nach Wissensgewinn könnte darauf zurückgeführt werden, dass sie sich zunächst intellektuell befähigt fühlen möchten, bevor sie sich am Dialog beteiligen. Die Abgrenzung zwischen Public Understanding und Public Engagement könnte daher nicht als Dichotomie, sondern als Kontinuum verstanden werden.

Wenngleich sich das Format in seiner Konzeption eher am Public Engagement-Paradigma orientiert, ist das Verständnis von Wissenschaftskommunikation und das Publikumsbild der Kommunikator*innen im Sinne eines Public Understanding of Science geprägt (vgl. Mede und Schäfer 2020). In ihren Aussagen spiegelt sich das Spannungsfeld zwischen einem normativen Anspruch an Dialog und Partizipation und deren tatsächlicher Umsetzung im Rahmen des Debattenformats wider. Hier sind sich die Kommunikator*innen unschlüssig, wie „echte“ Beteiligung ermöglicht und was damit erreicht werden soll. Das mag damit zusammenhängen, dass Public Engagement viele Potenziale zugeschrieben werden, die noch nicht empirisch nachgewiesen sind, und dass nicht eindeutig ist, welche Formen von Wissenschaftskommunikation dazu zu zählen sind. Bislang liegen meist breit gefasste Definitionen vor: „Clearly, there is no entirely satisfactory definition of public engagement.“ (Bauer und Jensen 2011, S. 4)

Die Kommunikator*innen haben eine Stärkung der wissenschaftlichen Perspektive in gesellschaftlichen Diskursen und mündige, kritische Bürger*innen zum Ziel, die sich evidenzbasiert Meinungen bilden und in Wissenschaft vertrauen, was eine stark normative Haltung gegenüber Wissenschaftskommunikation widerspiegelt. Ihr Rollenverständnis bewegt sich zwischen „Engagement“ und „Educational“, sie möchten die Popularisierung von Wissenschaft fördern, verstehen sich aber auch als kritische Informationsvermittler*innen (vgl. Blöbaum 2008). Insgesamt bleiben die Ausführungen vielfach an der Oberfläche; die Verantwortlichen werden selten explizit, wie sie ihre Ziele konkret realisieren möchten. Welche Ansprüche das einerseits von ihnen als elitär, andererseits zugleich aber als wissensdefizitär wahrgenommene Publikum womöglich an das Format hat, findet wenig Eingang in ihre Überlegungen. Es zeigen sich Inkongruenzen in den Erwartungshaltungen. Eine Annäherung zwischen Praktiker*innen und ihren Formatnutzer*innen könnte darüber erfolgen, dass konkretisierte Ziele transparent und für das Publikum nachvollziehbar gemacht werden. Zukünftige Untersuchungen zu den multiplen Publika von Wissenschaftskommunikation können zudem das Verständnis von Kommunikator*innen erhöhen und ihnen ein reales Bild davon vermitteln, wer ihre Formate mit welchen Erwartungen nutzt. Zu diskutieren bleibt, inwieweit Kommunikator*innen Publikumsinteressen letztlich ernst nehmen und wie sie mit enttäuschten Erwartungen umgehen sollten (vgl. Görke 2014). Um hierbei evidenzbasiert argumentieren zu können, erscheint es wichtig, beispielsweise zu erforschen, ob Publikumseinschätzungen zukünftiges Informationsverhalten leiten (vgl. Wolling 2009). Deutlich wurde, dass eine Orientierung an normativen Modellen mit Publikumsansprüchen verbunden werden und sich eine Qualitätsbestimmung nicht nur auf angebotsbezogene Qualitätsindikatoren beziehen sollte. Es ist noch ein Aushandlungsprozess zwischen Praxis, Politik, Wissenschaft und Bevölkerung, an welchen normativen, gesellschaftlichen, ökonomischen oder organisatorischen Leitplanken die Beziehung von Wissenschaft und Öffentlichkeit ausgerichtet sein soll. Ein erster Ansatz dafür ist die Denkfabrik „#FactoryWisskomm“, ein vom Bundesministerium unlängst initiierter Strategieprozess (BMBF 2021), bei dem Vertreter*innen aus Wissenschaft und Kommunikation über die Zukunft der Wissenschaftskommunikation in Deutschland diskutiert haben.

Diese Studie unterliegt durch die Format‑, Themen- und Methodenwahl Einschränkungen. Sie konzentriert sich auf das populäre Format Expert*innendebatte. Neben Anforderungen, die sich generell auf den Austausch zwischen Wissenschaft und Öffentlichkeit bzw. die Kommunikation zwischen wissenschaftlichen Expert*innen und Nicht-Expert*innen beziehen, äußerten die Teilnehmenden und Praktiker*innen format- und situationsspezifische Erwartungen. Inwiefern sich die vorgelegten Befunde bzw. die Qualitätserwartungen auf andere Vermittlungsformen und Rezeptionssituationen übertragen lassen, bedarf daher weiterer format- und themenvergleichender Untersuchungen. Erkenntnisse zu gestalterischen und inhaltlichen Merkmalen, die dem Publikum als Zeichen von Qualität dienen, helfen, die Entwicklung geeigneter Qualitätsdimensionen und Messinstrumente fortzusetzen. Jüngere Studien zeigen beispielsweise die Relevanz von Online-Kommunikation als Bestandteil diversifizierter Informationsrepertoires und ihre positiven Effekte auf Wissen auf (vgl. z. B. Su et al. 2015, S. 607). In Anschlussstudien könnte das dem Live-Stream folgende Debattenpublikum vergleichend analysiert und in den Blick genommen werden, inwiefern sich die beiden Publika voneinander unterscheiden.

Die Untersuchung baut des Weiteren auf einer Debatte zum Thema Wohnungsmarkt auf. Es ist unklar, inwiefern die Befragten bei der Angabe ihrer Erwartungen das Thema und/oder Format reflektiert oder ihre generellen Qualitätsvorstellungen zur Vermittlung von Wissenschaft geäußert haben. Es ist anzunehmen, dass sich Qualitätshaltungen in einem gewissen Maß am Thema und beispielsweise dessen wahrgenommener Wissenschaftlichkeit, Komplexität und Alltagsnähe orientieren können. Eine Systematisierung und Abstrahierung von Themencharakteristika würde zukünftig eine erhöhte Vergleichbarkeit ermöglichen. Deren Einbettung in Abfragen kann zudem Aufschluss über die Themenwahrnehmung des Publikums geben. Da insbesondere die erhofften Eigenschaften von Expert*innen eher allgemeingültiger Natur und nicht nur spezifisch im Kontext eines Debattenformats zutreffend sind, kann dennoch von einer gewissen Verallgemeinerbarkeit der vorgelegten Ergebnisse ausgegangen werden.

Neben Format und Thema kann sich ebenso das methodische Design auf die vorgelegten Befunde ausgewirkt haben. Da die Teilnehmenden *vor* der Rezeption der Debatte nach ihren Erwartungen gefragt wurden, achteten sie gegebenenfalls kritischer darauf, inwiefern diese erfüllt wurden, sodass die Qualitätsbewertung womöglich etwas negativer ausgefallen ist. Ihre Antworten können zudem von sozialer Erwünschtheit geprägt sein. Denkbar sind methodische Zugänge wie Echtzeitmessungen oder Post-Exposure-Walkthroughs, mit denen individuelle Urteilbildungsprozesse stärker rekonstruiert und erklärt werden können. So wäre es möglich, Qualitätsdimensionen gesondert zu analysieren und Faktoren zu ermitteln, die zu einer positiven oder negativen Beurteilung führen. Da diese nicht ausschließlich von den „Eigenschaften des Wertungsobjektes“ (Serong 2015, S. 13) abhängt, sondern auch von den Anforderungen derjenigen, die diese Eigenschaften beurteilen, können in Zukunft anhand repräsentativer Stichproben individuelle, soziodemographische Einflussfaktoren stärker einbezogen werden (vgl. Arnold 2009). Ebenso könnte sich die Forschung vermehrt denjenigen zuwenden, die bereits unzufrieden und desinteressiert sind (vgl. Humm et al. 2020), um deren Abwendung von Wissenschaft entgegenzuwirken. Unklar ist, inwiefern u. a. Themeninteresse, Vorwissen und Einstellungen, persönliche Betroffenheit, Informationsverhalten oder Involvement die Erwartungen und Bewertungen beeinflussen. Entsprechend könnte die Theorie der subjektiven Qualitätsauswahl erweitert werden, die das Individuum dahingehend bislang nicht integriert.

Eine vertiefende Beforschung der Sichtweisen von Kommunikator*innen ist ebenfalls lohnenswert. Ihre (impliziten) Vorstellungen über Erfolg und Ziele ihrer Formate prägen die Konzeptionierung und Weiterentwicklung wissenschaftskommunikativer Aktivitäten maßgeblich. Von diesen wird heute weitaus mehr erwartet, als wissenschaftliches Wissen der Öffentlichkeit zugänglich zu machen (vgl. Jensen und Gerber 2020, S. 2). Die vorliegende Studie zeigt diesbezüglich, dass das Publikum neben einer inhaltlichen Qualität auch eine gewisse „Erlebnisqualität“ erwartet, sodass von Wissenschaftskommunikator*innen eine Vielzahl an Rollen und Fähigkeiten gefordert ist – von der kuratorischen Aufgabe der Inhaltsaufbereitung bis hin zur*m Eventmanager*in. Was „gute“ Wissenschaftskommunikation charakterisiert, wie diese evidenzbasiert bestimmt und in der Praxis umgesetzt werden kann, erfordert nicht zuletzt weitere Auseinandersetzungen in der kommunikationswissenschaftlichen Forschung, denn, wie ein*e Kommunikator*in konstatiert: „Ganz schön kompliziert, gute Wissenschaftskommunikation zu machen!“ (K2).

## Anhang

### Pre-Post-Fragebogen (Auszug)

**Table 4:**

Dimension	Item Pre-Fragebogen (Erwartungen)	*n*	M	*SD*	Item Post-Fragebogen (Wahrnehmungen und Bewertungen)	*n*	M	*SD*
*1) Funktional-systemorientierte Ebene*								
Vielfalt	In der Expert*innendebatte sollte/n … auch mal andere als die üblichen Expert*innen mit ihren Positionen zu Wort kommen	40	4,18	*0,96*	Der Expert*innendebatte ist es gelungen, dass … auch mal andere Experten mit ihren Positionen zu Wort gekommen sind	35	3,46	*1,12*
	Bei einer Expert*innendebatte ist mir wichtig, dass … ich ein Thema aus unterschiedlichen Blickwinkeln kennenlerne	40	4,65	*0,83*	Der Expert*innendebatte ist es gelungen, dass … ich ein Thema aus unterschiedlichen Blickwinkeln kennengelernt habe	40	3,53	*0,72*
	In der Expert*innendebatte sollte/n … verschiedene wissenschaftliche Perspektiven deutlich werden	40	4,18	*0,71*	In der Expert*innendebatte wurde/n … verschiedene wissenschaftliche Perspektiven deutlich	40	3,50	*0,88*
Aktualität	Bei einer Expert*innendebatte ist mir wichtig, dass … das behandelte Thema aktuell ist	40	3,50	*1,22*	Der Expert*innendebatte ist es gelungen, dass … das behandelte Thema aktuell war	40	4,45	*0,78*
	Eine Expert*innendebatte, die meinen Vorstellungen entspricht, ist: aktuell	39	3,77	*1,20*	Die Expert*innendebatte zum Thema Wohnungsmarkt war: aktuell	40	4,58	*0,64*
Relevanz	Bei einer Expert*innendebatte ist mir wichtig, dass … nur relevante Aspekte des jeweiligen Themas angesprochen werden	40	3,80	*1,09*	Der Expert*innendebatte ist es gelungen, dass … nur relevante Aspekte des jeweiligen Themas angesprochen wurden	39	3,38	*0,91*
	Eine Expert*innendebatte, die meinen Vorstellungen entspricht, ist: relevant	39	4,26	*0,91*	Die Expert*innendebatte zum Thema Wohnungsmarkt war: relevant	40	4,25	*0,84*
Glaubwürdigkeit	Wie wichtig ist es Ihnen, dass ein/e Experte/in der Expert*innendebatte über folgende Eigenschaften verfügt? glaubwürdig	40	4,58	*0,68*	Wie haben Sie die Expertin/den Experten wahrgenommen?^a^ glaubwürdig	40	4,33	*0,49*
	Bei einer Expert*innendebatte ist mir wichtig, dass … Aussagen und Inhalte glaubwürdig sind	40	4,58	*0,78*	Der Expert*innendebatte ist es gelungen, dass … Aussagen und Inhalte glaubwürdig waren	40	4,15	*0,83*
	Eine Expert*innendebatte, die meinen Vorstellungen entspricht, ist: faktenbasiert	40	4,73	*0,64*	Die Expert*innendebatte zum Thema Wohnungsmarkt war: faktenbasiert	39	3,95	*0,76*
Unabhängigkeit	Von einer guten Expert*innendebatte erwarte ich, dass … die inhaltliche Themenbehandlung von Geldgeber*innen und Förderern unbeeinflusst ist	40	4,43	*0,87*	Der Expert*innendebatte ist es gelungen, dass … die inhaltliche Themenbehandlung von Geldgeber* innen und Förderern unbeeinflusst geblieben ist	34	3,88	*1,04*
	Wie wichtig ist es Ihnen, dass ein/e Experte/in der Expert*innendebatte über folgende Eigenschaften verfügt? unabhängig	40	4,33	*0,97*	Wie haben Sie die Expertin/den Experten wahrgenommen?^a^ unabhängig	40	4,02	*0,64*
Recherche	Von einer guten Expert*innendebatte erwarte ich, dass … im Vorfeld die Hintergründe der Expert*innen sorgfältig recherchiert wurden	39	3,95	*0,89*	In der Expert*innendebatte zum Thema Wohnungsmarkt … wurden im Vorfeld die Hintergründe der Expert*innen sorgfältig recherchiert	39	4,13	*0,98*
	Wie wichtig ist es Ihnen, dass ein/e Experte/in der Expert*innendebatte über folgende Eigenschaften verfügt? inhaltlich gut vorbereitet	40	4,48	*0,68*	Wie haben Sie die Expertin/den Experten wahrgenommen?^a^ inhaltlich gut vorbereitet	40	4,37	*0,50*
Kritik	Wie wichtig ist es Ihnen, dass ein/e Experte/in der Expert*innendebatte über folgende Eigenschaften verfügt? kritisch	40	4,28	*0,75*	Wie haben Sie die Expertin/den Experten wahrgenommen?^a^ kritisch	40	3,70	*0,70*
Zugänglichkeit	Wie wichtig ist es Ihnen, dass ein/e Experte/in der Expert*innendebatte über folgende Eigenschaften verfügt? verständlich	40	4,48	*0,72*	Wie haben Sie die Expertin/den Experten wahrgenommen?^a^ verständlich	40	4,04	*0,55*
	Eine Expert*innendebatte, die meinen Vorstellungen entspricht, ist: verständlich	40	4,48	*0,68*	Die Expert*innendebatte zum Thema Wohnungsmarkt war: verständlich	40	4,05	*0,64*
	In der Expert*innendebatte sollte/n … komplexe wissenschaftliche Inhalte verständlich erklärt werden	40	4,23	*0,86*	In der Expert*innendebatte wurde/n … komplexe wissenschaftliche Inhalte verständlich erklärt	40	3,15	*1,08*
*2) Normativ-demokratieorientierte Ebene*								
Ausgewogenheit	Wie wichtig ist es Ihnen, dass ein/e Experte/in der Expert*innendebatte über folgende Eigenschaften verfügt? abwägend	39	3,77	*1,01*	Wie haben Sie die Expertin/den Experten wahrgenommen?^a^ abwägend	39	3,59	*0,65*
	Bei einer Expert*innendebatte ist mir wichtig, dass … das Thema ausgewogen und umfassend debattiert wird	40	4,35	*0,70*	Der Expert*innendebatte ist es gelungen, dass … das Thema ausgewogen und umfassend debattiert wurde	39	3,36	*0,99*
	Eine Expert*innendebatte, die meinen Vorstellungen entspricht, ist: ausgewogen	39	3,90	*0,94*	Die Expert*innendebatte zum Thema Wohnungsmarkt war: ausgewogen	38	3,63	*0,88*
	In der Expert*innendebatte sollte/n … die Unsicherheit wissenschaftlicher Erkenntnisse thematisiert werden	40	4,00	*0,93*	In der Expert*innendebatte wurde/n … die Unsicherheit wissenschaftlicher Erkenntnisse thematisiert	37	2,16	*1,04*
Neutralität	Wie wichtig ist es Ihnen, dass ein/e Experte/in der Expert*innendebatte über folgende Eigenschaften verfügt? objektiv	39	4,36	*0,87*	Wie haben Sie die Expertin/den Experten wahrgenommen?^a^ objektiv	40	3,86	*0,49*
Achtung der Persönlichkeit	Wie wichtig ist es Ihnen, dass ein/e Experte/in der Expert*innendebatte über folgende Eigenschaften verfügt? respektvoll	36	4,00	*0,97*	Wie haben Sie die Expertin/den Experten wahrgenommen?^a^ respektvoll	40	4,52	*0,49*
*3) Nutzerbezogen-handlungsorientierte Ebene*								
Anwendbarkeit	Wie wichtig ist es Ihnen, dass ein/e Experte/in der Expert*innendebatte über folgende Eigenschaften verfügt? hilfreich	36	3,69	*0,98*	Wie haben Sie die Expertin/den Experten wahrgenommen?^a^ hilfreich	40	3,58	*0,77*
	Bei einer Expert*innendebatte ist mir wichtig, dass … mir konkrete Handlungsempfehlungen gegeben werden	39	3,36	*1,04*	Der Expert*innendebatte ist es gelungen, dass … mir konkrete Handlungsempfehlungen gegeben wurden	39	2,23	*1,14*
	Eine Expert*innendebatte, die meinen Vorstellungen entspricht, ist: hilfreich	40	3,60	*0,93*	Die Expert*innendebatte zum Thema Wohnungsmarkt war: hilfreich	40	2,88	*0,91*
	In der Expert*innendebatte sollte/n … ein Alltagsbezug von wissenschaftlicher Forschung aufgezeigt werden	40	3,78	*0,77*	In der Expert*innendebatte wurde/n … ein Alltagsbezug von wissenschaftlicher Forschung aufgezeigt	38	2,79	*1,09*
Unterhaltsamkeit	Eine Expert*innendebatte, die meinen Vorstellungen entspricht, ist: unterhaltsam	40	3,03	*1,10*	Die Expert*innendebatte zum Thema Wohnungsmarkt war: unterhaltsam	39	2,15	*0,88*
	Wie wichtig ist es Ihnen, dass ein/e Experte/in der Expert*innendebatte über folgende Eigenschaften verfügt? unterhaltend	36	3,14	*1,25*	Wie haben Sie die Expertin/den Experten wahrgenommen?^a^ unterhaltend	39	2,51	*0,91*
*4) Wissenschaftskommunikations- bzw. formatspezifische Aspekte*								
Bildung, Aufklärung und Information	Eine Expert*innendebatte, die meinen Vorstellungen entspricht, ist: informierend	40	4,40	*0,67*	Die Expert*innendebatte zum Thema Wohnungsmarkt war: informierend	40	3,73	*0,88*
	In der Expert*innendebatte sollte/n … vermittelt werden, wie wissenschaftliche Erkenntnisse gewonnen werden	39	3,59	*1,09*	In der Expert*innendebatte wurde/n … vermittelt, wie wissenschaftliche Erkenntnisse gewonnen werden	39	2,15	*1,07*
	Von einer guten Expert*innendebatte erwarte ich, dass … ich etwas über das debattierte Thema dazulerne	40	4,60	*0,67*	In der Expert*innendebatte zum Thema Wohnungsmarkt … konnte ich etwas über das debattierte Thema dazulernen	40	4,03	*0,89*
	Wie wichtig ist es Ihnen, dass ein/e Experte/in der Expert*innendebatte über folgende Eigenschaften verfügt? informierend	35	4,31	*0,83*	Wie haben Sie die Expertin/den Experten wahrgenommen?^a^ informierend	40	4,00	*0,59*
Legitimation und Akzeptanz	In der Expert*innendebatte sollte/n … die Bedeutung von Wissenschaft für die Gesellschaft deutlich werden	39	3,46	*1,12*	In der Expert*innendebatte wurde/n … die Bedeutung von Wissenschaft für die Gesellschaft deutlich	39	2,54	*1,21*
Dialog und Partizipation	Eine Expert*innendebatte, die meinen Vorstellungen entspricht, ist: dialogorientiert	39	3,54	*0,97*	Die Expert*innendebatte zum Thema Wohnungsmarkt war: dialogorientiert	40	3,20	*1,14*
	Wie wichtig ist es Ihnen, dass das Publikum in die Expert*innendebatte einbezogen wird?	39	2,90	*1,23*	Wie gut wurde Ihrer Meinung nach das Publikum in die Debatte einbezogen?	39	3,33	*1,06*
	Von einer guten Expert*innendebatte erwarte ich, dass … ich Expert*innen Fragen stellen und meine Meinung mitteilen kann	40	3,05	*1,20*	In der Expert*innendebatte zum Thema Wohnungsmarkt … konnte ich Expert*innen Fragen stellen und meine Meinung mitteilen	40	3,73	*1,01*
	Wie wichtig ist es Ihnen, dass ein Experte der Expert*innendebatte über folgende Eigenschaften verfügt? dialogbereit/-interessiert	36	4,06	*0,86*	Wie haben Sie die Expertin/den Experten wahrgenommen?^a^ dialogbereit/-interessiert	39	3,72	*0,79*
Emotionen	Wie wichtig ist es Ihnen, dass ein/e Experte/in der Expert*innendebatte über folgende Eigenschaften verfügt? leidenschaftlich	35	3,29	*1,41*	Wie haben Sie die Expertin/den Experten wahrgenommen?^a^ leidenschaftlich	40	2,82	*0,88*
	Bei einer Expert*innendebatte ist mir wichtig, dass … mich das Thema emotional anspricht	39	2,97	*1,18*	In der Expert*innendebatte zum Thema Wohnungsmarkt … hat mich das Thema emotional angesprochen	40	2,75	*1,26*
	Eine Expert*innendebatte, die meinen Vorstellungen entspricht, ist: emotional	40	2,73	*1,36*	Die Expert*innendebatte zum Thema Wohnungsmarkt war: emotional	40	1,80	*0,76*
Rahmenbedingungen	Von einer guten Expert*innendebatte erwarte ich, dass … sie gut organisiert ist und z. B. die Technik (Mikrophone, Licht) einwandfrei funktioniert	40	4,00	*0,91*	Der Expert*innendebatte ist es gelungen, dass … sie gut organisiert war und z. B. die Technik (Mikrophone, Licht) einwandfrei funktioniert hat	40	4,08	*0,97*
*Weitere Items*								
–	–				Wie gut hat Ihnen insgesamt die Expert*innendebatte zum Thema Wohnungsmarkt gefallen?	40	3,48	*0,75*
					Würden Sie erneut eine Expert*innendebatte aus der Veranstaltungsreihe besuchen?	39	3,85	*1,09*
					Würden Sie die Expert*innendebatte weiterempfehlen?	40	3,55	*0,93*

Fünfstufige Skala von 1 = „stimme überhaupt nicht zu“/„überhaupt nicht wichtig“/„sehr schlecht“/„nein, auf gar keinen Fall“ bis 5 = „stimme voll und ganz zu“/„sehr wichtig“/„sehr gut“/„ja, unbedingt“

^a^Im Post-Fragebogen jeweils für die drei Expert*innen gesondert erhoben, hier als Summenindex zusammengefasst

### Leitfaden (Auszug)

**Table 5:**

*Thematischer Block: Wissenschaftskommunikation*	
Was ist Ihre Rolle bzw. Position in Ihrer Institution?	–
Was ist das Selbstverständnis Ihrer Institution?	–
Wenn man an die Beziehung von Wissenschaft und Öffentlichkeit denkt, wird diese häufig anhand der Entwicklung des Paradigmas Public Understanding of Science hin zum Public Engagement of Science diskutiert. Was bedeutet Ihrer Meinung nach „Public Understanding of Science“ bzw. „Public Engagement with Science“?	Wie sollte Wissenschaftskommunikation sein? Welche Erwartungen haben Sie an die Vermittlung von Wissenschaft? Könnten Sie Ihr persönliches Verständnis von Wissenschaftskommunikation auf eine solche Formel bringen?
Denken Sie, dass auf Seiten der Bürger*innen ein Beteiligungsbedürfnis besteht?	Welche Form von Beteiligung von Bürger*innen stellt einen Mehrwert dar? Welche Probleme könnten aber auch damit verbunden sein?
Wen erreicht Wissenschaftskommunikation? Welchen Eindruck, welches Bild haben Sie von wissenschaftsfernen bzw. -nahen Bürger*innen?	Ist ein ‚Dialog auf Augenhöhe‘ mit Bürger*innen (überhaupt) möglich?
Wie sollten Wissenschaftsthemen in der Öffentlichkeit thematisiert werden?	Wie sollte Ihrer Meinung nach mit wissenschaftlicher Unsicherheit, konfligierenden Erkenntnissen, kontroversen Positionen etc. umgegangen werden? Inwiefern sollte das in den Medien/in der Öffentlichkeit thematisiert werden? Und wie können bzw. sollen Bürger*innen damit umgehen?
Was bedeutet (verlässliche) wissenschaftliche Expertise? Wer ist ein Experte?	–
Welche Rolle/welche Funktionen sollte Wissenschaftskommunikation einnehmen, bspw. im Zusammenhang mit Politik?	Inwiefern sollte Wissenschaftskommunikation beratend agieren oder sich (wert-)neutral verhalten?
Was charakterisiert Ihrer Meinung nach ‚gute‘ Wissenschaftskommunikation?	–
*Thematischer Block: Hintergrund des Projektes und der Formatentwicklung*	
Was ist der Mehrwert von dem Format und inwiefern unterscheidet es sich von bisherigen Formaten?	Was ist der Innovationswert? Was ist das Besondere? Wieso braucht es dieses Format? Wieso erachten Sie den ‚Einsatz‘ von Expert*innen als sinnvoll?
Inwiefern sollen Bürger*innen eingebunden und an der Debatte beteiligt werden?	Welche Interaktionsformen soll es aus welchen Gründen geben? Wie interaktiv soll es sein? Im Sinne eines Public Understanding oder Engagement?
Was erwarten Sie, wie die Debatten vom Publikum wahrgenommen werden?	–
*Thematischer Block: Publikumsbild und Zielgruppe*	
Wen möchten Sie mit dem Format erreichen und weshalb?	Was wäre ein ‚typischer‘ Teilnehmer? An wen denken Sie? Wie schätzen Sie das Themeninteresse und Wissen derjenigen ein, die an den Veranstaltungen vor Ort teilnehmen?
Was könnten Teilnahme-Motive sein?	Was bringt den Teilnehmenden dazu, zu der Veranstaltung zu gehen?
Welche (weitere) Zielgruppe kann mit der Website und dem Online-Stream erreicht werden?	Inwiefern unterscheiden sich die Online-Nutzenden von denjenigen, die an Präsenzveranstaltungen teilnehmen?
Wie hoch schätzen Sie generell das Interesse von Bürger*innen an Wissenschaft bzw. wissenschaftlichen Themen ein?	Woran mag das liegen?
*Thematischer Block: Ziele des Formats*	
Welche Themen sollen im Rahmen der Debatte behandelt werden? Wieso?	Wie findet eine Auswahl statt? Welche Themen sprechen Bürger*innen an? Wieso?
Wie sollen die Themen aufbereitet werden?	Welche Ziele sollen dabei verfolgt werden? Inwiefern geht es um eine verständliche Vermittlung, um eine wissenschaftlich korrekte Aufbereitung, um einen Alltagsbezug, um Meinungsbildung etc.?
Was möchten Sie mit dem Format bei den Teilnehmenden erreichen?	Welche ‚Veränderungen‘ könnte eine Teilnahme bewirken, bspw. hinsichtlich Interesse und Verständnis für wissenschaftliche Prozesse, Scientific Literacy, Einstellungen gegenüber den Themen?

## Footnotes

1 Zur Herleitung, Begründung und Beschreibung der einzelnen Kriterien siehe Arnold (2009, S. 162–224).
